# Accuracy and Precision of a Veterinary Neuronavigation System for Radiation Oncology Positioning

**DOI:** 10.1155/2018/6431749

**Published:** 2018-02-13

**Authors:** Isabelle F. Vanhaezebrouck, Elizabeth A. Ballegeer, Stephen Frey, Rob Sieffert

**Affiliations:** ^1^Small Animal Clinical Sciences, College of Veterinary Medicine, Michigan State University, 736 Wilson Road, East Lansing, MI 48824, USA; ^2^Rogue Research Inc., 6666 St-Urbain, Suite 300, Montreal, QC, Canada H2S 3H1

## Abstract

Conformal radiation treatment plans such as IMRT and other radiosurgery techniques require very precise patient positioning, typically within a millimeter of error for best results. CT cone beam, real-time navigation, and infrared position sensors are potential options for success but rarely present in veterinary radiation centers. A neuronavigation system (Brainsight Vet, Rogue Research) was tested 22 times on a skull for positioning accuracy and precision analysis. The first 6 manipulations allowed the authors to become familiar with the system but were still included in the analyses. Overall, the targeting mean error in 3D was 1.437 mm with SD 1.242 mm. This system could be used for positioning for radiation therapy or radiosurgery.

## 1. Introduction

Precise positioning is essential for the success of radiation treatment. Any potential error in set-up will jeopardize the efficacy of the treatment, leading to less control of the local cancer and risking irradiation of healthy tissue.

The International Commission of Radiation Unit (ICRU) in Report 50 [1] defines the gross tumor volume (GTV) as the tumor evaluated by palpation and/or image analysis. The clinical target volume (CTV) refers to the GTV in addition to the margins surrounding the tumor due to microscopic disease. The planned target volume (PTV), based on the CTV, encompasses errors due to organs movement, set-up errors, or equipment imprecision. These volumes are assessed by the radiation oncologist. CT or MRI scans are essential for a radiation dose planned to the cancer area with the intent to minimize the dose to other critical structures.

The identification of the margins around tumor volumes has been honed with technological progress such as conformation devices (intensity modulated radiation therapy, or IMRT) and image guidance. Today with certain types of treatment in radiosurgery (tumor treatment with one large dose of radiation only, or three to five sessions for stereotactic radiation therapy), margins have been reduced to a minimum of 1 mm or less for the radiation of intracranial structures [2]. Fine collimation devices such as lead cones, 120 multileaf collimators, or modern software programs, such as intensity modulated radiation therapy, arc therapy, or volumetric modulated arc therapy, have allowed very successful conformal radiation plans. The final result, however, will depend on the linear accelerator precise delivery and correct patient positioning.

Manual plans are set up after a visual evaluation (skin) and/or palpation of bony protuberances (less mobile), while computerized plans benefit from image guidance. For the irradiation of intracranial structures, the usage of port films improves set-up accuracy. A systematic 3D error of 2.4–3.1 mm for patient positioning has been reported with a standard deviation of 2 mm [3]. Patient heads are immobilized; lasers aligned on marks and port films are used for verification. In 95% of cases in the human radiation field, as well as the veterinary field, there is a 3D error less than 5-6 mm with this type of imaging guidance [3, 4]. A small number of veterinary radiation centers are equipped with OBI (On Board Imaging) cone beam scanner. This is an advantage for the evaluation in 3 dimensions, providing the easiest evaluation of patient rotation and better assessment of internal organs. It has been shown that 1 mm of error or less can be achieved in targeting accuracy and precision for intracranial radiation set-up (for 3D vectors) with the assistance of OBI technique positioning [5].

Infrared camera tracking in radiation oncology has been validated by the human neurosurgeon Meeks et al. [6] and it is an option for precise positioning.

Tracking is the process of measuring the location of instruments, anatomical structures, and/or landmarks in relationship to each other in the 3-dimensional (3D) space of the patient. With passive tracking, a position sensor emits a signal reflected by infrared light emitting diodes (IRLED) and four diodes (minimum) are fixed on the patient. Light reflection from the diodes is processed by software (Fast Plan ND –/Varian, Inc., 3100 Hansen Way, Paolo Alto, CA, USA) and translated into the patient position in 3D. Numerical values of *x*, *y*, *z*, yaw, pitch, and roll are sent to a computer workstation that help the therapist place the patient in the initial position with the assistance of a reference tracker. Patients are positioned within 1 mm of error and a rotational shift less than one degree [7]. There are no reports in the veterinary literature of such procedures.

Recently, Washington State University College of Veterinary Medicine has described a minimally invasive technique for canine brain biopsy with neuronavigation tracking system guidance. The accuracy of the system with this procedure was verified with postsurgical MRI scans (mean error to target 1.79 ± 0.87 mm). All injections were realized with less than 3.31 mm error [8].

We hypothesized that we could use the veterinary neurosurgical device for our radiation set-up on patients. We set out to check if the accuracy and the precision would be sufficient for future hypofractionated radiation treatments, including radiosurgery for intracranial tumors.

## 2. Materials and Methods

**Figure 1:**
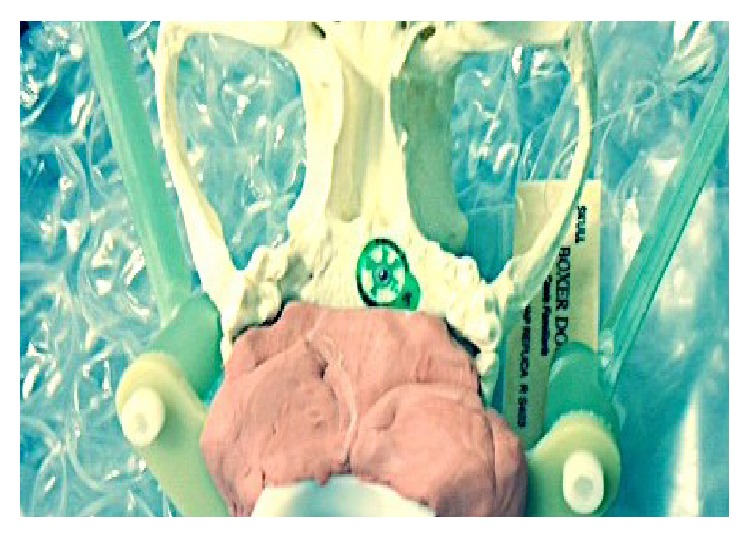
Fiducial on the skull.

**Figure 2:**
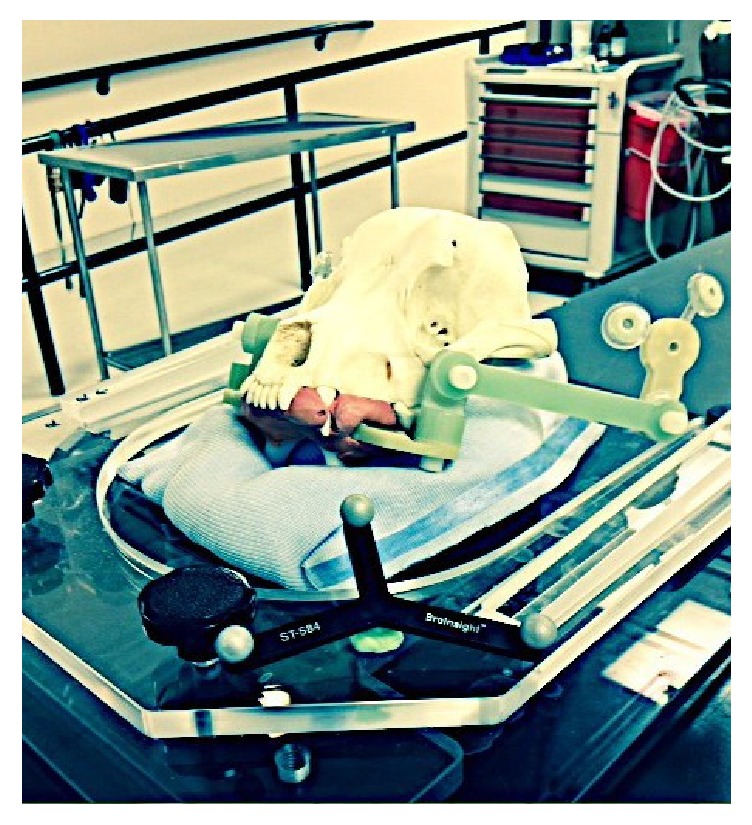
Bite block, subject tracker (IRLED diodes), and radiation cushion.

A 1 mm metallic fiducial (target center) was positioned randomly on a canine skull. A bite block made from dental composite and four IRLED diodes were attached to the skull (Figures 1 and 2). The same skull was used for each positioning trial.

**Figure 3:**
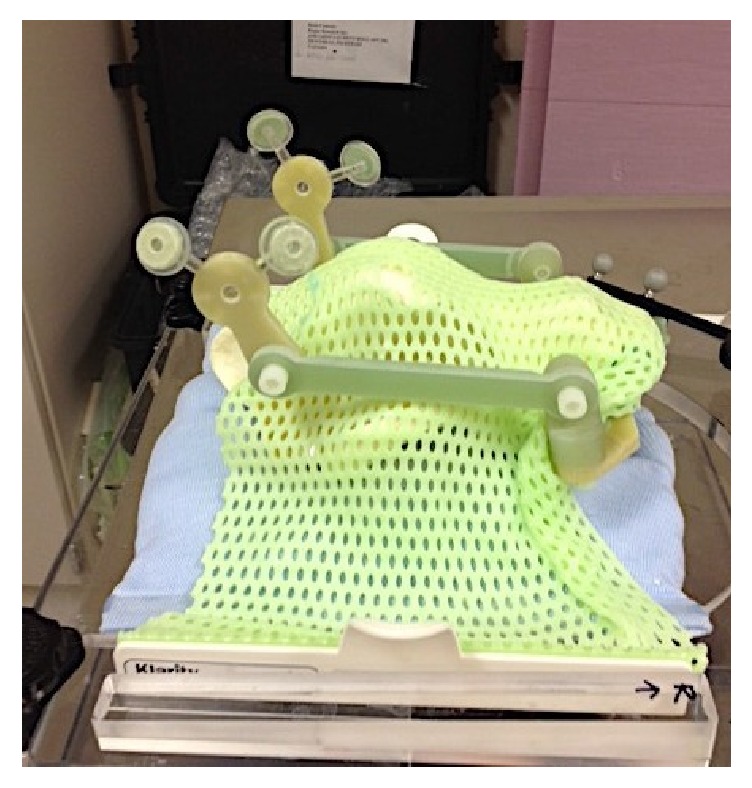
Thermoplastic mask.

The skull sat on a moldable pillow for head and neck radiation (Moldcare ND) and was covered with a thermoplastic mask (Klarity ND) (Figure 3). Most veterinary radiation facilities use a bite block and a thermoplastic mask on their patients for precise head and neck positioning.

**Figure 4:**
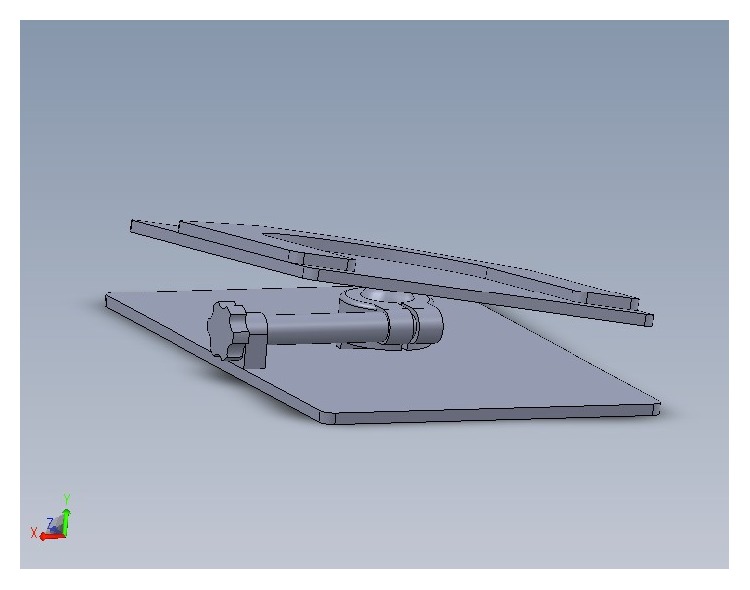
Engineered base for precise final set-up.

The full system was then laid on an engineered base plate with fine movements possible in 6 directions (*X*, *Y*, *Z*, yaw, pitch, and roll) (Figure 4).

With laser guidance in the LINAC room, two opposed ink marks (one lateral and one dorsal) were placed on the thermoplastic mask surface. This defined future CT scanner reference positions.

**Figure 5:**
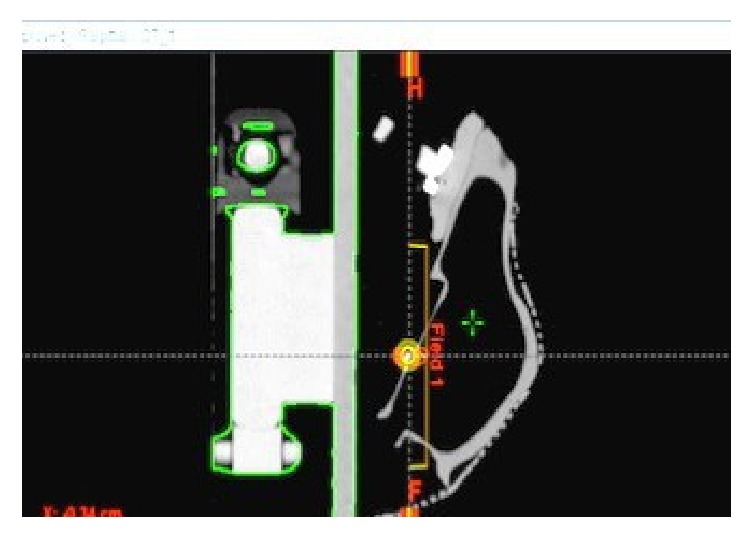
Mask CT reference (green cross) versus fiducial (gold dot)/shift treatment plan.

The phantom skull was transported to radiology for CT imaging (GE® Brightspeed 16-slice helical scanner, GE Medical, Milwaukee, WI), and the CT reference positions were registered. DICOM images were then transferred to the radiation treatment plan (Eclipse ND) and the Brainsight software (Rogue Research). The metallic fiducial was selected as the center of the tumor and the iso-center of radiation fields for the phantom. Shifts between the CT reference and the fiducial's exact position were obtained from the radiation treatment plan (Eclipse ND) (Figure 5) and then forwarded to the neuronavigation software Brainsight (Rogue Research).

**Figure 6:**
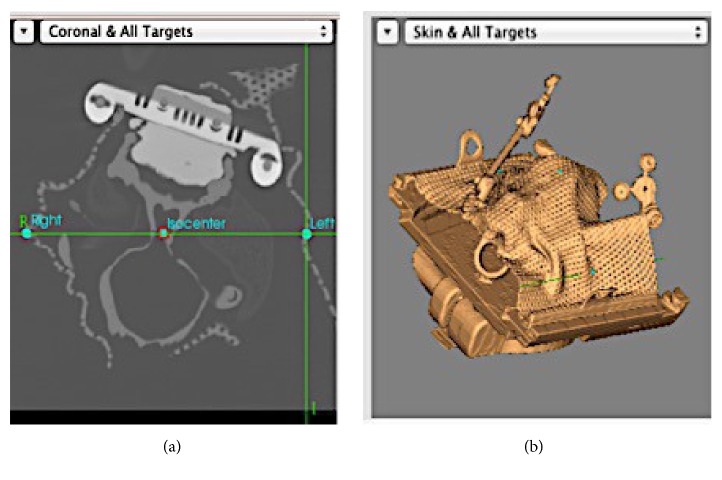
Isocenter (fiducial) location with the neuronavigation software.

The phantom was registered to the scans using the neuronavigation software, and three vectors (one dorsal, two laterals) were drawn from the hypothetical tumor location (Figures 6(a) and 6(b)).

**Figure 7:**
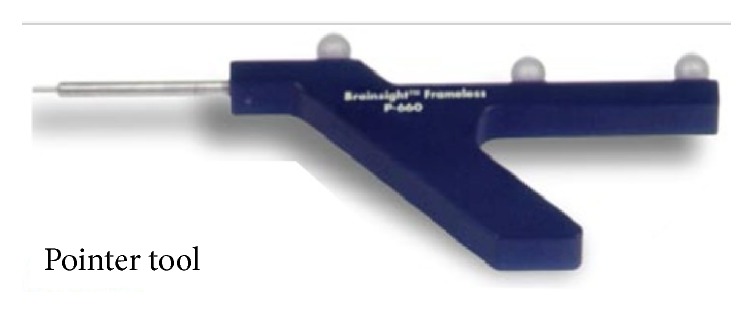
Neuronavigation pointer Courtesy Rogue Research.

**Figure 8:**
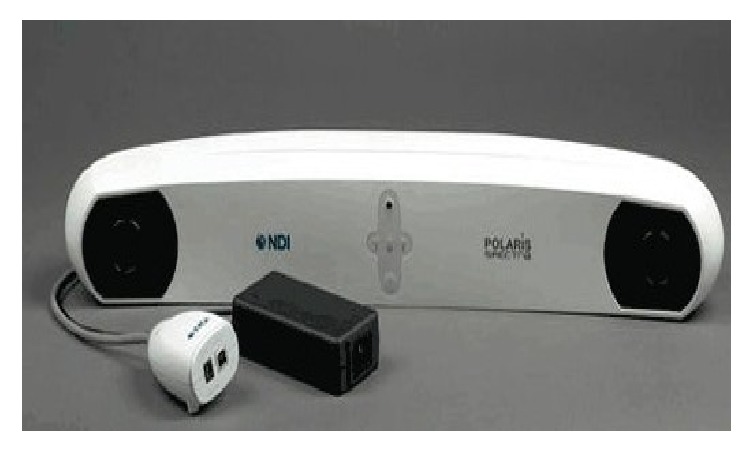
Vicra Position Sensor, Courtesy Northern Digital Inc.

For each trial, the skull was reassembled (bite plate insertion, diodes, mask, and cushion) in the radiation LINAC area, as it would be for a real patient set-up, before radiation treatment delivery. The IRLED camera and neuronavigation pointer were tested and the phantom was coregistered (Figures 7 and 8).

**Figure 9:**
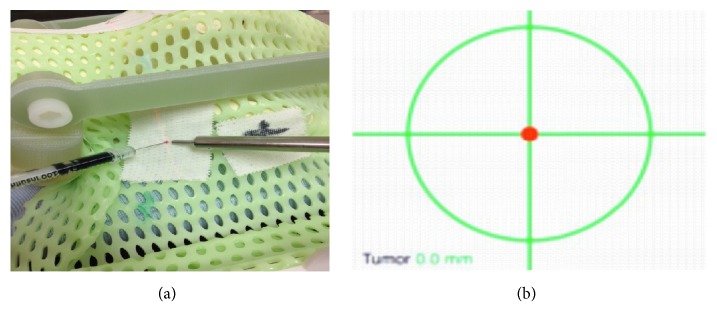
Final marks after Brainsight software guidance.

The four IRLED diodes were identified by the neuronavigation pointer, and the information was sent to the Brainsight software. The neuronavigation pointer assisted in finding the dorsal and lateral positions in relation to the fiducial location (where the traced vectors intersected with the skull surface, Figures 6(a) and 6(b)). Dots were marked and verified 3 times before final positioning (Figures 9(a) and 9(b)).

For final positioning, vault lasers are placed on the skull dots. The therapist or the radiation oncologist adjusted the couch translation and fine movements of the bite plate (yaw, pitch, and roll) (Figure 4).

**Figure 10:**
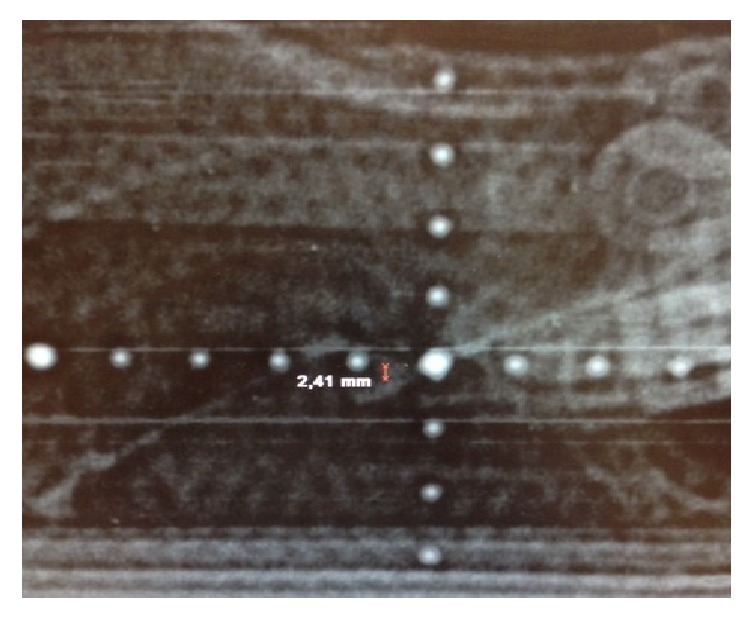
90 degrees or orthogonal port film.

**Figure 11:**
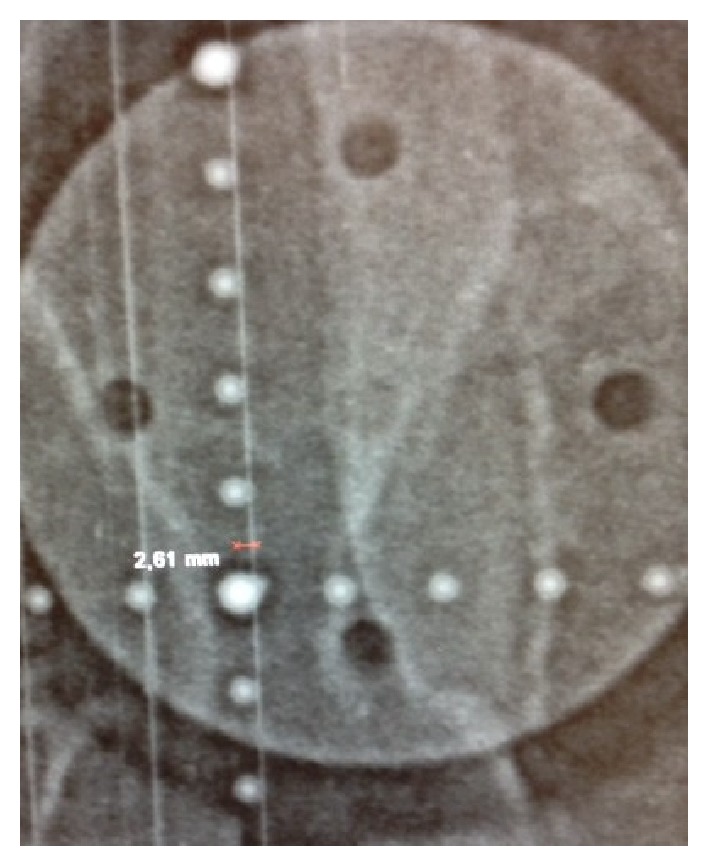
Dorsal port film.

Verification of the exact fiducial position was obtained with a dorsal and a 90-degree port film (Figures 10 and 11). The coordinates (*dx*, *dy*, and *dz*) from the “in room set-up” and the real fiducial position (recorded from the port films) were compared.

## 3. Results and Analysis

The same skull was used for 22 manipulations. For each experiment, the mask and the bite block were reinstalled. The skull was positioned and the fiducial position was tracked with the neuronavigation system. Verification of the exact fiducial position was processed after two portal films. The translation error for each direction, *X*, *Y*, and *Z*, was reported and separated into systematic versus random error.

The systematic error is represented by the mean deviation and evaluates accuracy. The random error is the difference between the mean deviation and the total error and represents precision. 2D and 3D vectors were calculated.

(1) d3D=dx2+dy2+dz2,d2D=dy2+dz2.

**Figure 12:**
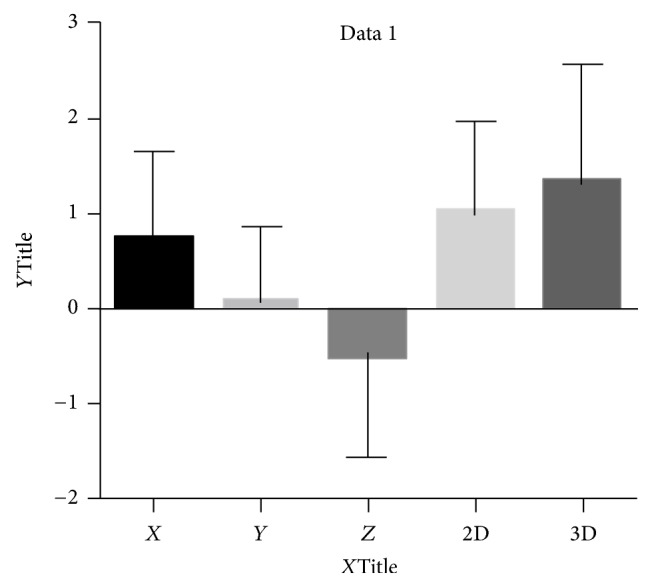
Data distribution.

Tables 1, 2, and 3 and Figure 12 report the descriptive statistics for twenty-two manipulations, deviation *dx*, *dy*, and *dz*, mean, and standard deviation.

According to these authors, it took 6 manipulations (experiments) to become comfortable with the navigation system and the set-up.

**Figure 13:**
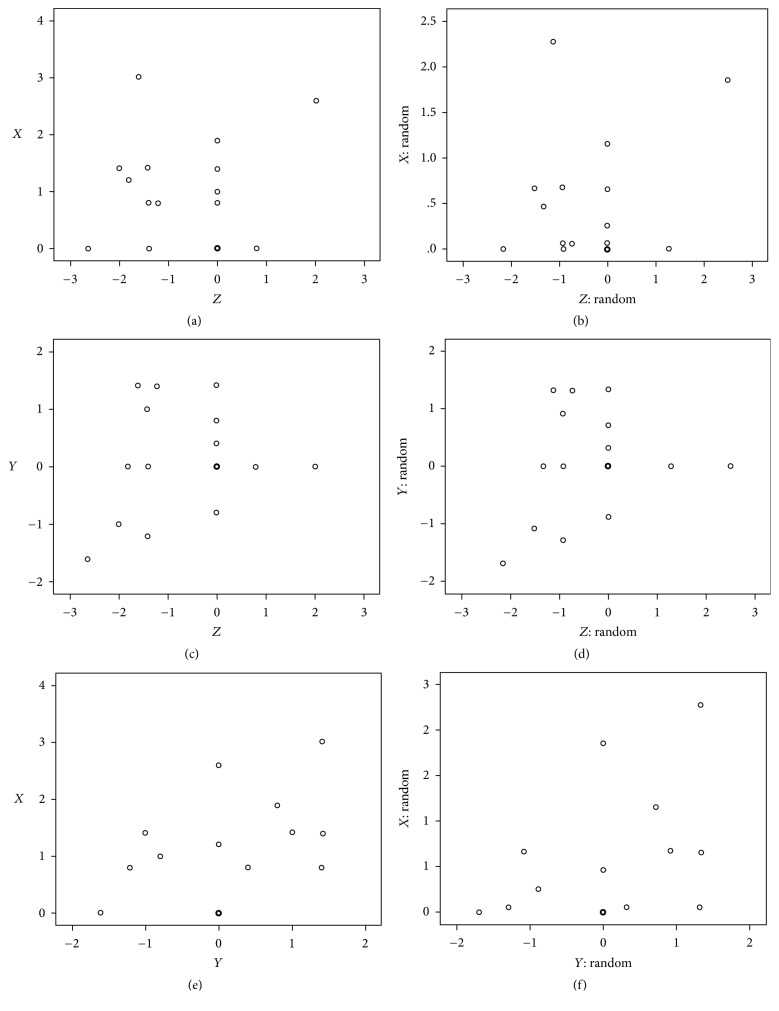
Scatters plots absence of correlations between errors on different axes (systematic and random).

- The 2D mean vector was 1 mm, SD was 0.969 mm, and the 3D mean vector was 1.3 mm, and SD was 1.242 mm, with less than 1 mm accuracy (3D mean is 0.919 mm and SD is 1.097 mm if we discard the 6 first manipulations based on practice).
- There was no correlation observed between the different axes errors (Figures 13(a)–13(f), square plots).

## 4. Study Limits

The physicist verified the vault laser positions before the first experiment. We elected the thinnest slice thickness possible on our available 16-detector row CT scanner, 0.6 mm. Thinner and more precise LINAC lasers exist and could improve our results.

The analysis is dependent on the quality of our port films as well as the resolution of our analysis software; the port films (pictures from the megavoltage linear accelerator) are digitalized by a scanner, and distances are measured with an electronic caliper. A KV imager (X-ray generator separately mounted on the LINAC) would optimize this process (Figures 10 and 11). Having more independent inter- and intraraters could possibly increase the statistical power.

On the radiation couch, we noticed that repeating the registration procedure for finding the fiducial position 3 times increased reliability of positioning before final assignment of position. Furthermore, the Brainsight pointer necessitates steady hands and there is jitter from the infrared position sensor, which can lead to error. An insulin needle with ink instead of a blunt-pointed permanent marker delivers better precision and accuracy on the marks as we discovered in the beginning (Figure 9(b)).

The position camera is allowed to warm up before patient registration is important. The temperature inside the room needs to be checked as thermal drift has been reported with some IRLED cameras. It is for this reason that the camera was powered on 20 minutes before manipulations.

We have used two different types of dental composites. The 3M (Express™ VPS Impression Material) provided better reproducibility for the bite plate set-up than the putty Reprosil™ (Dentsply International Inc.).

## 5. Conclusion

Twenty-two experiments were carried out on a single canine skull with the guidance of the IRLED Brainsight (Rogue Research) neuronavigation system which led to an accuracy of 1.347 mm (SD = 1.242 mm) to target. If we discard the first 6 registrations (learning period) on the phantom skull, the precision increased to 0.919 mm and SD is 1.097 mm. Submillimeter lasers inside the linear accelerator bunker and a KV imager with fine software resolution would have optimized our results.

With practice, the entire process takes less than 10 minutes for set-up in the LINAC. This positioning product can serve both neurologists and radiation oncologists in a specialty veterinarian center. We are confident that it could be of help for the veterinary radiation oncologist in getting precision and accuracy for patients benefiting from radiosurgery treatments.

## Figures and Tables

**Table 1:** Descriptive statistics.

Data (mm)	*X* + right	*Y* + in	*Z* + up

	0	−1.61	−2.64
	3.02	1.41	−1.61
	1.21	0	−1.81
	1.41	−1	−2
	0.8	−1.21	−1.41
	1	−0.8	0
	0	0	0
	0	0	0
	0	0	−1.4
	0	0	0.8
	0	0	0
	1.4	1.42	0
	0.8	1.4	−1.22
	1.9	0.8	0
	2.6	0	2.02
	0	0	0
	0.8	0.4	0
	0	0	0
	1.42	1	−1.42
	0	0	0
	0	0	0
	0	0	0
Mean	0.74364	0.08227	−0.48591
Standard deviation	0.91737	0.79617	1.06496

**Table 2:** Descriptive statistics: 2D, 3D vectors.

Measure	Mean	Median	SD	Skew	SE	Kurtosis	SE
*X*	0.744	0.400	0.917	1.101	0.491	0.504	0.953
*Y*	0.082	0.000	0.796	−0.080	0.491	0.192	0.953
*Z*	−0.486	0.000	1.065	−0.032	0.491	0.303	0.953
*X*: random	0.372	0.028	0.638	2.033	0.491	3.657	0.953
*X*: systematic	0.372	0.372	0.381	0.000	0.491	−2.211	0.953
*Y*: random	0.045	0.000	0.792	−0.267	0.491	0.310	0.953
*Y*: systematic	0.037	0.000	0.042	0.196	0.491	−2.168	0.953
*Z*: random	−0.265	0.000	0.962	0.809	0.491	2.616	0.953
*Z*: systematic	−0.221	0.000	0.248	−0.196	0.491	−2.168	0.953
*d*_3D_	1.347	1.340	1.242	0.292	0.491	−1.203	0.953
*d*_2D_	1.017	0.800	0.969	0.385	0.491	−1.054	0.953

**Table 3:** Non-Gaussian distribution.

One-sample Kolmogorov-Smirnov tests		
Measure	Test statistic	*p*
*X*	.291	<.001
*Y*	.277	<.001
*Z*	.312	<.001
D3D	.224	.005
D2D	.217	.009
*X*: random	.326	<.001
*X*: systematic	.336	<.001
*Y*: random	.296	<.001
*Y*: systematic	.359	<.001
*Z*: random	.301	<.001
*Z*: systematic	.359	<.001
